# Factorial Trial to Optimize an Internet-Delivered Intervention for Sexual Health After Breast Cancer: Protocol for the WF-2202 Sexual Health and Intimacy Enhancement (SHINE) Trial

**DOI:** 10.2196/57781

**Published:** 2024-08-19

**Authors:** Kelly M Shaffer, Jennifer Barsky Reese, Emily V Dressler, Jillian V Glazer, Wendy Cohn, Shayna L Showalter, Anita H Clayton, Suzanne C Danhauer, Michelle Loch, Mai Kadi, Caleigh Smith, Kathryn E Weaver, Glenn J Lesser, Lee M Ritterband

**Affiliations:** 1 University of Virginia School of Medicine Charlottesville, VA United States; 2 Fox Chase Cancer Center Philadelphia, PA United States; 3 Wake Forest University School of Medicine Winston-Salem, NC United States; 4 Louisiana State University Health Sciences Center New Orleans, LA United States

**Keywords:** breast neoplasms, cancer survivorship, clinical trial, communication skills, factorial trial, internet interventions, intimacy, mobile phone, multiphase optimization strategy, sexual distress, sexual functioning, sexual health

## Abstract

**Background:**

Although most survivors of breast cancer report substantial sexual concerns following treatment, few receive support for these concerns. Delivering sexual health care to survivors of breast cancer via the internet could overcome many of the barriers to in-person treatment. Even when delivered remotely, survivor time constraints remain a leading barrier to sexual health intervention uptake.

**Objective:**

Guided by the multiphase optimization strategy methodological framework, the primary objective of this study is to identify the most efficient internet-delivered sexual health intervention package that is expected to provide survivors of breast cancer the greatest benefit with the fewest (and least-intensive) intervention components. This study aims to determine how intervention components work (mediators) and for whom they work best (moderators).

**Methods:**

Partnered, posttreatment adult female survivors of breast cancer (N=320) experiencing at least 1 bothersome sexual symptom (ie, pain with sex, vaginal dryness, low sexual desire, and difficulty with orgasm) related to their breast cancer treatment will be enrolled. Clinic-based recruitment will be conducted via the Wake Forest National Cancer Institute Community Oncology Research Program (NCORP) Research Base. Participants will be randomly assigned to 1 of 16 combinations of four intervention components with two levels each in this factorial trial: (1) psychoeducation about cancer-related sexual morbidity (receive either enhanced vs standard versions); (2) communication skills training for discussing concerns with health care providers (received vs not received); (3) communication skills training for discussing concerns with a partner (received vs not received); and (4) intimacy promotion skills training (received vs not received). Cores will be fully automated and implemented using a robust internet intervention platform with highly engaging elements such as animation, video, and automated email prompts. Survivors will complete web-based assessments at baseline (prerandomization time point) and again at 12 and 24 weeks later. The primary study aim will be achieved through a decision-making process based on systematically evaluating the main and interaction effects of components on sexual distress (Female Sexual Distress Scale–Desire, Arousal, Orgasm) and sexual functioning (Female Sexual Function Index) using a generalized linear model approach to ANOVA with effect coding. Mediation analyses will be conducted through a structural equation modeling approach, and moderation analyses will be conducted by extending the generalized linear model to include interaction effects.

**Results:**

This protocol has been reviewed and approved by the National Cancer Institute Central Institutional Review Board. Data collection is planned to begin in March 2024 and conclude in 2027.

**Conclusions:**

By identifying the combination of the fewest and least-intensive intervention components likely to provide survivors of breast cancer the greatest sexual health benefit, this study will result in the first internet intervention that is optimized for maximum impact on the undertreated, prevalent, and distressing problem of breast cancer–related sexual morbidity.

**Trial Registration:**

ClinicalTrials.gov NCT06216574; https://clinicaltrials.gov/study/NCT06216574

**International Registered Report Identifier (IRRID):**

PRR1-10.2196/57781

## Introduction

### Background

Breast cancer and its treatment can cause physical (eg, loss of body part and vaginal dryness), psychological (eg, stress and body dissatisfaction), and relational concerns (eg, restricted communication and relational discord) that can lead to problems with sexual distress and dysfunction [1,2]. Therefore, approximately 70% of survivors of breast cancer report clinically relevant symptoms of sexual distress and dysfunction [2-5], which they rank among their most distressing cancer-related problems [6,7]. These sexual concerns are particularly severe among women who were premenopausal at diagnosis, as treatments can induce menopause with more severe symptoms [8-10]; take adjuvant endocrine therapy (AET; tamoxifen or aromatase inhibitors) as it blocks estrogen [11-13]; have comorbid depression and anxiety [14-16]; and have newer relationships or are generally more dissatisfied in their relationship [5,17-19]. Unlike psychological or informational needs that tend to remit naturally, survivors’ unmet sexual health needs *increase* over time [20]. Given the prevalence and profound impacts of cancer on sexual health, oncology care standards recommend that all survivors receive information, assessment, and care for sexual symptoms [21,22]. Despite these recommendations, <1 in 3 survivors of breast cancer recall ever discussing sexual health with their cancer care team [23]. Thus, there is an urgent need to develop highly scalable interventions to support survivors’ sexual health.

Commonly cited reasons for the lack of comprehensive sexual health care within oncology include limited access to specialty services, provider and patient discomfort in discussing sexual health, and appointment time constraints [24,25]. Delivering fully automated care via the internet can help alleviate these barriers to care by providing evidence-based education and behavioral strategies privately and on demand, anywhere an internet connection exists [26-28]. Comprehensive sexual health interventions have been demonstrated as effective when delivered remotely; however, the substantial time requirements of these multicomponent programs have been a leading barrier to enrolling and retaining survivors in care [28-30]. Maximizing the population-wide impact of sexual health interventions for survivors of breast cancer will require minimizing intervention requirements, without compromising the overall intervention efficacy. More efficient interventions may attract and retain more survivors to benefit from care.

A major gap in the literature regarding the efficacy of individual sexual health components has limited the development of more efficient sexual health treatments for survivors of breast cancer [29]. Because of the complex biopsychosocial nature of sexual health, interventions to improve sexual health after cancer typically include multiple therapeutic components. Leading clinical oncology organizations recommend that women are educated about the effects of cancer on their sexual response cycle, as well as interventions to improve intimacy and relationship concerns [21,31]. Communication skills training is critical to help survivors overcome the barriers of stigma to advocate for their sexual health needs [1]. Because interventions tested in this area to date have tended to test multiple components packaged together and in differing combinations across trials, it has been impossible to differentiate which components work, establish why those components work, or identify for whom different components work best. Addressing these gaps will help promote the development of more efficient, effective, and personalized interventions.

### Objectives

The overarching goal of our research is to develop a highly scalable and efficient breast cancer–related sexual health intervention with a strong potential for population-wide impact. Toward that goal, the primary aim of this trial is to identify the combination of the fewest intervention components likely to provide survivors of breast cancer with the greatest sexual health benefit. Intervention components to be tested are (1) level of education about the influence of breast cancer on sexual health, (2) communication skills training for discussing sexual concerns with one’s health care team, (3) communication skills training for discussing sexual concerns with one’s intimate partner, and (4) intimacy promotion skills training. These components, which address the complex biopsychosocial nature of sexual concerns after breast cancer, were selected based on the research team’s prior qualitative and intervention development research [32,33] and are consistent with survivorship care guidelines [21,22]. This aim will be achieved through evaluating the impact of each component, alone and in combination, on survivors’ sexual distress and functioning.

**Figure 1:**
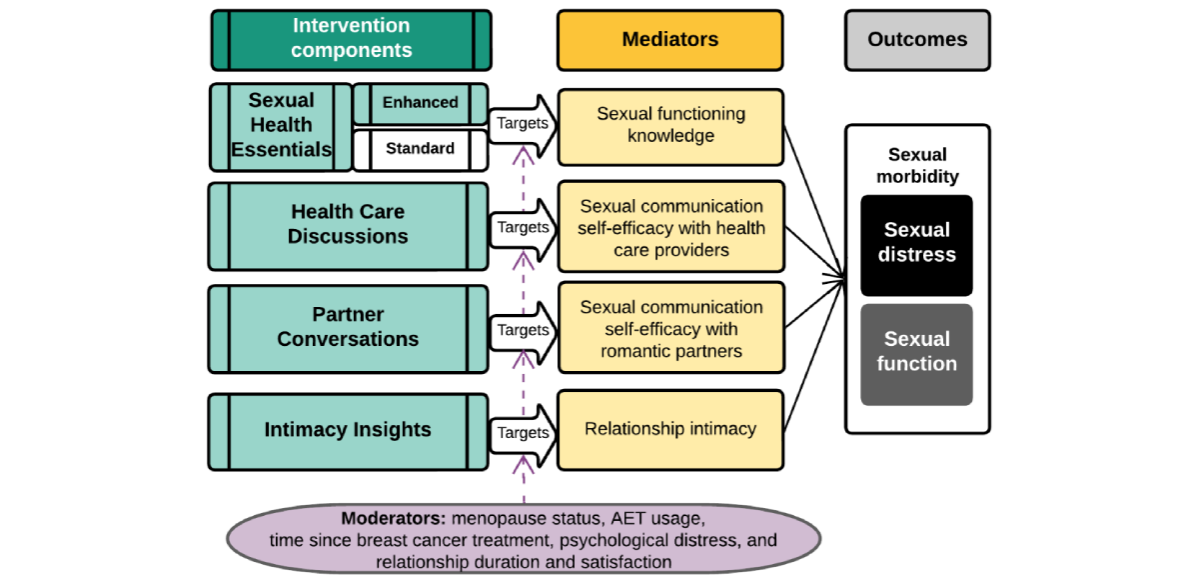
Study conceptual model. AET: adjuvant endocrine therapy.

This trial will also evaluate the clinical mechanisms of the intervention components and whether different groups of survivors may need different combinations of intervention components. We hypothesize that the intervention components will influence sexual morbidity via mediators selected based on social cognitive theory [34], specifically, through increased knowledge about sexual concerns and self-efficacy to discuss them, as well as emotional intimacy with one’s partner (see Figure 1 for the trial conceptual model). We also hypothesize that a priori moderators, selected according to clinical and psychosocial characteristics known to influence sexual morbidity in survivors of breast cancer, will influence the components’ efficacy. Achieving these research objectives will improve cancer survivorship care by establishing the first internet intervention for breast cancer–related sexual morbidity that has been optimized for maximum impact, as well as clarifying how intervention components work and for whom they work best.

## Methods

### Ethical Considerations

The study protocol has been reviewed and approved by the National Cancer Institute (NCI) Central Institutional Review Board (CIRB; WF-2202 initially approved on December 1, 2023). Study modifications will be reviewed and approved by the NCI CIRB. Participating study sites and their local institutional review boards will be notified of any modifications. Deidentified data sets used in primary publications will be transferred to the National Clinical Trials Network and NCI Community Oncology Research Program (NCORP) Data Archive in accordance with the agreement between Wake Forest (WF) NCORP Research Base (RB) and NCI. The internet intervention platform hosting the Sexual Health and Intimacy Enhancement (SHINE) intervention is managed by the University of Virginia (UVA) and was designed to support projects that contain protected health information that are subject to compliance with federal and state regulations. Access to data will be in accordance with the Health Care Financing Administration’s Internet Security Policy and the Health Insurance Portability and Accountability (HIPAA) Act of 1996. The database servers and web servers are backed up daily and are protected from threats by regular maintenance and the application of hotfix and security patches when released. Security policies are in place with UVA to ensure that access to database repositories and web application server environments is limited. On completion of the trial, summary results will be submitted to ClinicalTrials.gov in accordance with National Institutes of Health (NIH) policies [35]. No participant identifiers will be published.

During the informed consent process, potential participants are informed that this study aims to determine whether an internet-delivered program can help survivors of breast cancer manage cancer-related sexual concerns. Under a waiver of documentation of consent, participants will provide either signed informed consent or verbal informed consent that is documented by the enrolling research staff member. The NCI CIRB–approved informed consent form will be made available open-access with other study materials. Participants will receive a US $50 e-gift card for completing the 12-week assessment and a US $75 e-gift card for completing the 24-week assessment. On request at the end of the 24-week assessment, participants will be provided access to all the SHINE program components through the end of the grant period.

### Trial Design

This factorial trial will enroll 320 adult survivors of breast cancer with sexual concerns to test the effects of 4 sexual health intervention components. The primary trial objective is to determine the optimal breast cancer–related sexual health intervention package. The secondary objective is to examine mediators and moderators of intervention component efficacy, and we will also explore survivors’ intervention engagement and acceptability. This manuscript reports the protocol for the WF-2202 SHINE trial following recommendations from the 2013 SPIRIT (Standard Protocol Items: Recommendations for Interventional Trials) [36,37]; Table 1 provides the World Health Organization trial registration data set. The study protocol was revised following the NIH peer review process (see Multimedia Appendix 1 for reviews).

**Table 1:** World Health Organization trial registration data set.

Data category			Information
Primary registry and trial identifying number			ClinicalTrials.gov identifier: NCT06216574
Date of registration in primary registry			January 10, 2024
Secondary identifying numbers			R37CA269776—National Institutes of Health grant; WF^a^-2202—WF NCI^b^ Community Oncology Research Program Research Base protocol; NCI-2023-06866—NCI Clinical Trials Reporting Program registry identifier
Sources of monetary or material support			NCI (grant UG1CA189824 [multiple principal investigators GJL and KEW] and R37CA269776 [principal investigator KMS]) The NCI provided a review of the study design. The NCI CIRB^c^ serves as the single IRB^d^ for this trial. The NCI will review publications before their submission.
Primary sponsor			University of Virginia Sponsors (primary and secondary) have no significant role in the design of this study and will not have any role during its execution, analyses, interpretation of the data, or decision to submit the results.
Secondary sponsors			WF University Health Sciences; Fox Chase Cancer Center
Contact for public queries and scientific queries			KMS, PhD, Center for Behavioral Health and Technology, UVA, Charlottesville, Virginia (corresponding author)
Public title			SHINE^e^ study
Scientific title			WF-2202 Optimizing psychosocial intervention for breast cancer–related sexual morbidity
Countries of recruitment			The United States
Health conditions or problems studied			Breast cancer; sexual dysfunction
Interventions			SHINE components (see Multimedia Appendix 2 for intervention details): Sexual Health Essentials: receive either enhanced or standard versions of the Core Health Care Discussions: receive the Core or not Partner Conversations: receive the Core or not Intimacy Insights: receive the Core or not
Inclusion criteria			Ages eligible for study: ≥18 years Sexes eligible for study: female only Accepts healthy volunteers: no From medical record review: History of stage 0-III breast cancer diagnosis. History of nonbreast malignancies are permitted. ≥12 weeks following last primary cancer treatment. For this protocol, primary cancer treatments are defined as chemotherapy, cytotoxic antibody-drug conjugates, checkpoint inhibitors, radiation, and surgical procedures intended to remove malignant tissue. Ongoing AET^f^ (eg, tamoxifen, aromatase inhibitors), adjuvant CDK^g^ 4/6-inhibitors (eg, abemaciclib), HER2^h^-based monoclonal antibody therapy (eg, trastuzumab, pertuzumab), HER2 targeted tyrosine kinase inhibitors (eg, neratinib), and pending breast reconstructive surgery are allowed. Aged ≥18 years at the time of study enrollment From self-reported web-based screener Cisgender female (ie, assigned female at birth, female gender identity) Currently in an intimate relationship with an individual of any sex and gender identity Endorse being at least “somewhat” bothered by ≥1 of the following during the last 30 days: (lack of) interest in sexual activity, vaginal dryness, pain during sexual activity, or (lack of) ability to orgasm, as reported on the Patient-Reported Outcomes Measurement Information System SexFS Bother Regarding Sexual Function screener [38] Endorse that ≥1 of the bothersome sexual symptoms (from above criterion) is related to their breast cancer Has a working email address (or willing to create one) and receive emails from the study
Exclusion criteria			From medical record review Planned cancer treatment for residual, progressive, or recurrent disease within the 24 weeks following enrollment (defined as chemotherapy, cytotoxic antibody-drug conjugates, checkpoint inhibitors, radiation, and surgical procedures intended to remove malignant tissue). Ongoing AET (eg, tamoxifen, aromatase inhibitors), adjuvant CDK 4/6-inhibitors (eg, abemaciclib), HER2-based monoclonal antibody therapy (eg, trastuzumab, pertuzumab), HER2 targeted tyrosine kinase inhibitors (eg, neratinib), and pending breast reconstructive surgery are allowed. From self-reported web-based screener Cannot read and comprehend English as indicated by not completing the self-reported screening questionnaire independently No reliable access to the internet (eg, by home broadband, public network, personal data plan) by computer, tablet, smartphone, etc and is not willing to participate in the tablet lending program for this study Recent serious mental illness, as defined by reporting an inpatient psychiatric hospitalization within the past 12 months Currently participating in couple, marital, or sex therapy Currently pregnant
Study type			Interventional allocation: randomized Masking: investigator, outcomes assessor Assignment: factorial (2^4^) Primary purpose: supportive care
Date of first enrollment			Anticipated in March 2024
Target sample size			320
Recruitment status			Pending (as of manuscript submission)
Primary outcome			FSDS-DAO^i^: change from baseline to 24-week assessment
Key secondary outcomes			FSDS-DAO: 12-week assessment Female Sexual Function Index: 12- and 24-week assessments
Ethics review			NCI CIRB serves as the single IRB for this trial. Protocol WF-2202 received initial approval as of December 1, 2023. The study protocol will be reviewed and approved by the local IRB of each participating recruitment site. Participants will receive contact information from the local IRB for inquiries.
**Additional SPIRIT^j^ information**			
	Confidentiality	Data collected through self-report and medical record extraction is collected and maintained through REDCap^k^, a HIPAA^l^-compliant web-based data management tool. Participants’ data are saved with a unique participant identifier code; personally identifying information is saved separately. SHINE program use data are collected through the research team’s HIPAA-compliant internet intervention platform. No participant-identifying information will be shared outside the research team. Data are protected with a certificate of confidentiality	
	Protocol version	Version 01.04.2024; issue date: approved February 2, 2024; protocol amendment number: amendment 1; authors: KMS, Julie Turner	
	Nonapplicable items	Composition of the coordinating center and trial steering committee; criteria for discontinuing or modifying allocated interventions; procedure for unblinding if needed; interim analyses; additional consent provisions for collection and use of participant data and biological specimens; provisions for posttrial care; assignment of writing committees; plans for collection, laboratory evaluation and storage of biological specimens for genetic or molecular analysis in this trial or future use	

^a^WF: Wake Forest.

^b^NCI: National Cancer Institute.

^c^CIRB: Central Institutional Review Board.

^d^IRB: institutional review board.

^e^SHINE: Sexual Health and Intimacy Enhancement.

^f^AET: adjuvant endocrine therapy.

^g^CDK: cyclin-dependent kinase.

^h^HER2: human epidermal growth factor receptor 2.

^i^FSDS-DAO: Female Sexual Distress Scale–Desire, Arousal, Orgasm.

^j^SPIRIT: Standard Protocol Items: Recommendations for Interventional Trials.

^k^REDCap: Research Electronic Data Capture.

^l^HIPAA: Health Insurance Portability and Accountability Act.

Our study aims will be achieved via a factorial trial within the optimization phase of the multiphase optimization strategy (MOST) framework. The MOST methodological framework directly addresses our goal of optimizing a multicomponent intervention to improve sexual health after breast cancer [39,40]. In an optimization trial, the effects of intervention components are tested independently and in combination, which allows the identification of an efficient intervention package that only includes components shown to improve survivors’ sexual health. Components may also be evaluated to determine whether effects vary systematically across survivors, guiding future treatment tailoring. These insights directly address major research gaps regarding how sexual health intervention components work and for which survivors they work best [29].

**Figure 2:**
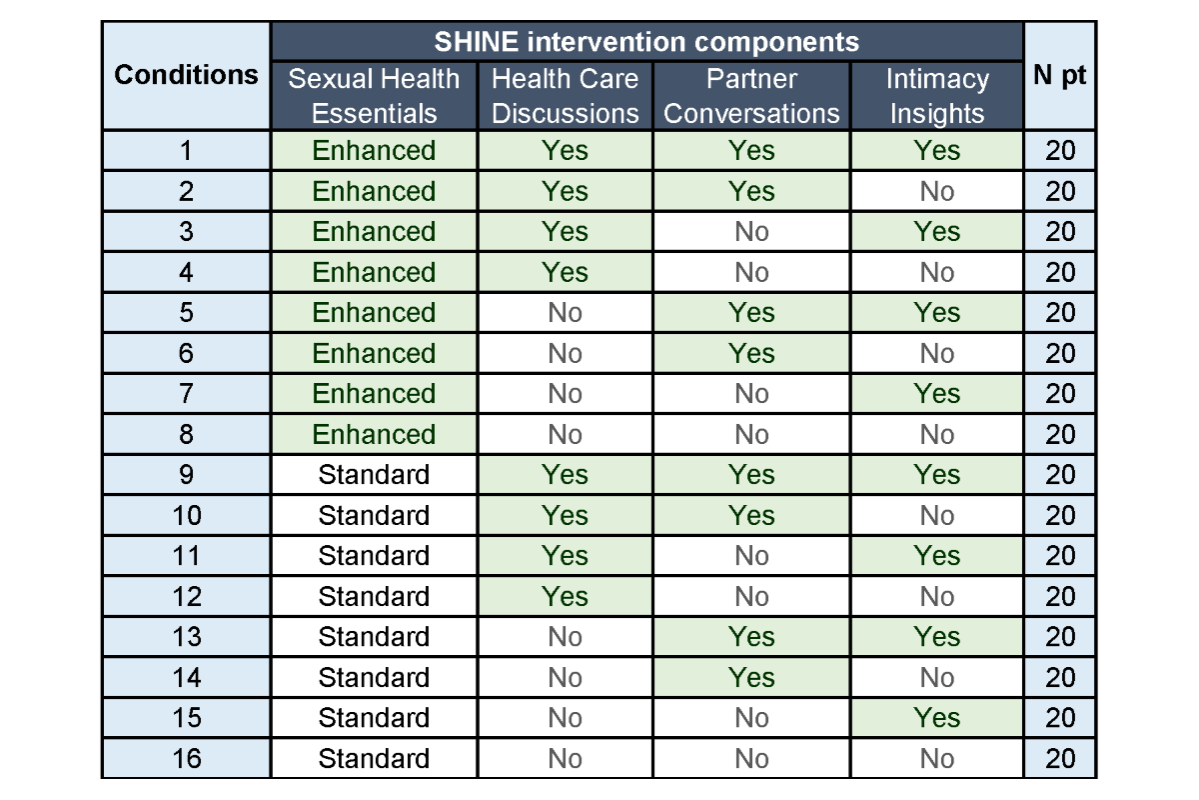
Study conditions. Pt: participants; SHINE: Sexual Health and Intimacy Enhancement.

In this factorial trial, participants will be randomized to 1 of 16 conditions in a factorial design involving 4 intervention components, each with 2 levels (2^4^; Figure 2). Participants may continue to receive standard care during the trial. The trial will be conducted via the WF NCORP RB, which has 42 affiliated health system sites, each with its own research infrastructure and dedicated research staff to identify, screen, consent, and enroll survivors for this trial. Only 10% of the affiliated sites are connected with an academic medical center, and 25% of sites are safety-net hospitals, serving patient populations that are commonly neglected in intervention development research [41]. The use of this oncology practice–based research network is important because it will both enhance the generalizability of results to diverse survivors treated in community settings and encourage the eventual translation of the optimized intervention into clinical practice. As required by the NCORP trials, the NCI CIRB serves as the institutional review board of record for this trial.

### Participants

This trial will enroll 320 survivors of breast cancer meeting the following inclusion criteria (see Table 1 for details): (1) history of stage 0 to III breast cancer diagnosis (history of other cancer diagnoses is permitted); (2) ≥12 weeks from last primary cancer treatment (eg, chemotherapy, surgery, or radiation therapy); (3) ≥18 years of age; (4) cisgender female; (5) in an intimate relationship with a person of any sex and gender; (6 and 7) endorse at least 1 bothersome sexual symptom that they attribute to their breast cancer; and (8) willing to receive emails from the study. Survivors will be excluded if they (1) have cancer treatment planned within the study period; (2) cannot read English; (3) do not have reliable internet access (and are not willing to participate in a study tablet loan program); (4) have had a recent episode of serious mental illness; (5) are in current couple, marital, or sex therapy; or (6) are currently pregnant. These eligibility criteria are in keeping with our prior breast cancer–related sexual health intervention research (eg, [42]) and support the evaluation of isolated intervention effects with less risk of contamination or confounding from other interventions or treatments.

### Procedures

**Figure 3:**
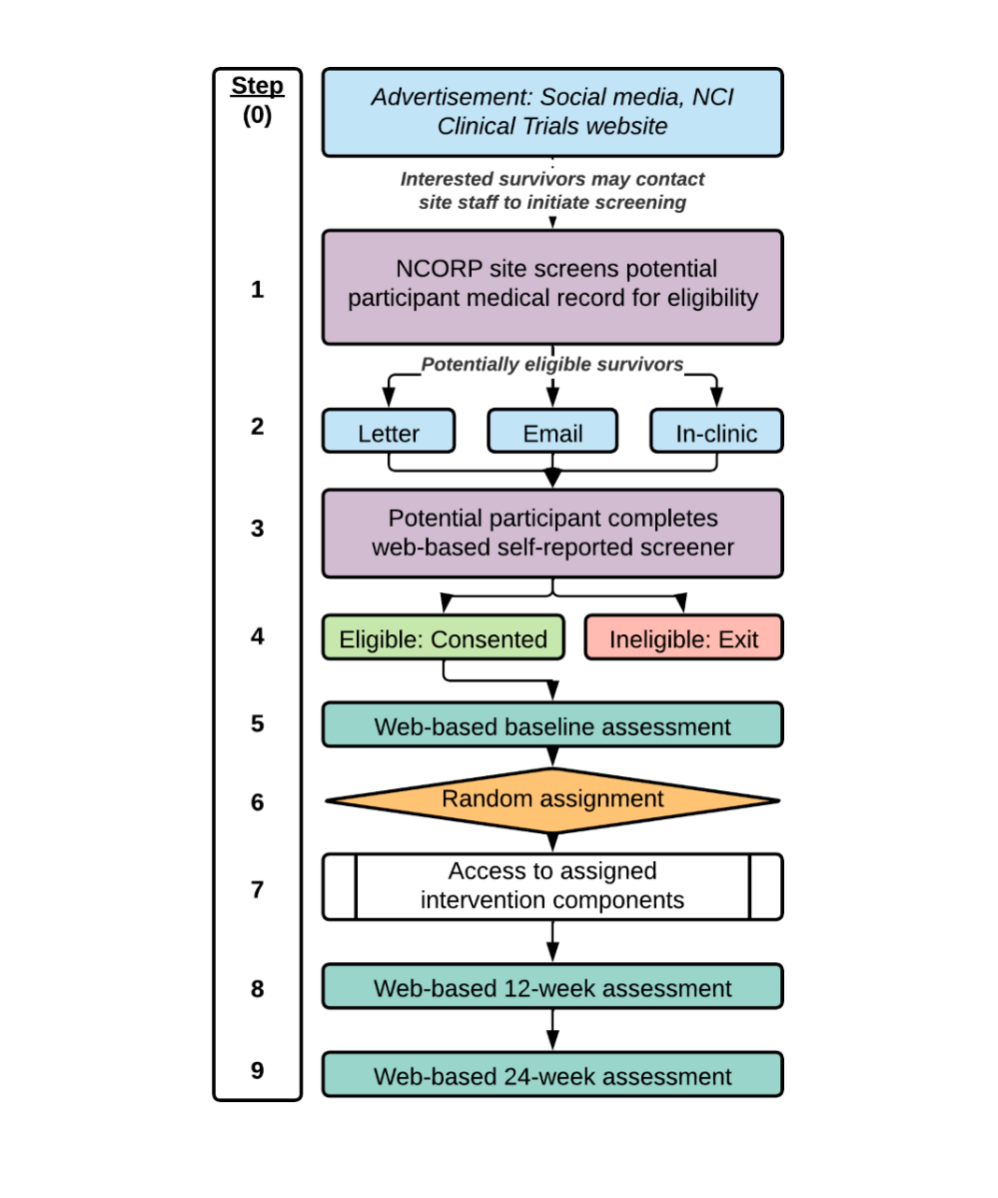
Study procedures. NCI: National Cancer Institute; NCORP: National Cancer Institute Community Oncology Research Program.

All study procedures, namely, recruitment, consenting, assessment, and intervention, can be completed remotely (eg, via the internet; see Figure 3 for a schematic of procedures). Potential participants’ medical records will be screened by research staff at the participating NCORP sites; this process may be initiated by the staff at the site or by a potentially interested participant contacting the site after learning of the trial via social media, other advertisement, or the NCI Clinical Trials page [43] (Figure 3, steps 0-1). Individuals who meet medical eligibility criteria will be contacted (step 2); those who are interested will complete a self-reported web-based screener regarding the remaining eligibility criteria (step 3). This screener will be completed through REDCap (Research Electronic Data Capture; Vanderbilt University), a HIPAA-compliant, web-based survey and research management platform. Individuals not meeting the eligibility criteria will be notified that they are ineligible for the trial, while those who are eligible will be contacted by the NCORP site staff to answer any remaining questions about the trial and obtain informed consent (step 4). Participants who do not have reliable access to the internet via computer, smartphone, or tablet will be offered a cellular data–enabled tablet to use for the full 24-week study period.

After enrollment, participants will be invited to complete the web-based baseline assessment via REDCap (step 5). On completion of the baseline assessment, participants will be automatically randomized to 1 of the 16 study conditions via REDCap (step 6). Block randomization will be used to help maintain balance across conditions, with varying block sizes of 16, 32, and 48 [44,45]. A participant’s randomized condition will not be revealed to the local NCORP study staff; only the UVA research coordinator (who implements participants’ conditions in the SHINE intervention system) and a WF NCORP RB data manager will have access to participants’ randomized condition information. Participants cannot be blinded to their condition. Participants will receive a welcome email directing them to set a password and log into their SHINE program (ie, the set of components assigned to them in their randomized condition; step 7).

After baseline, participants will be asked to complete their SHINE program within the following 12 weeks and to complete follow-up assessments through REDCap at 12 and 24 weeks (steps 8 and 9). A protocol of stepped reminder emails via REDCap and contacts by NCORP site research staff will encourage participants to complete all study assessments.

### Intervention

Intervention components are delivered fully automated as a *Core*, or lesson, of content. Components (and component levels) to be tested are (1) education about the influence of breast cancer on sexual health, either with limited information in a static web page format in the Sexual Health Essentials-Standard Core or more comprehensive information through an interactive web program in the Sexual Health Essentials-Enhanced Core; (2) communication skills training for discussing sexual concerns with one’s health care team in the Health Care Discussions Core; (3) communication skills training for discussing sexual concerns with one’s intimate partner in the Partner Conversations Core; and (4) intimacy promotion skills training in the Intimacy Insights Core. All participants will receive 1 of 2 Sexual Health Essentials Cores (ie, standard [component level=low] or enhanced [component level=high]) given that survivorship care guidelines recommend that all survivors receive education about the influence of cancer treatment on sexual health [21]. For the 3 remaining components, participants will either receive the Core (component level=on) or not receive the Core (component level=off; see Figure 2 for factorial design and Multimedia Appendix 2 for additional Core details).

The Sexual Health Essentials-Enhanced, Health Care Discussions, Partner Conversations, and Intimacy Enhancement Cores each deliver content as stand-alone, web-based programs. These Cores were developed following the Model for Internet Interventions [46] and user-centered design practices to be highly engaging, using interactive elements, audiovisuals, and tailoring. Cores were developed with a team of software engineers and consultants in user interface, user experience, and instructional design. Core content was refined through iterative feedback from survivors of breast cancer. The Sexual Health Essentials-Standard Core provides education via a traditional web page with no tailoring or interactive elements.

Cores take about 20-45 minutes to complete, which participants may do at their convenience and own pace (ie, Cores do not need to be completed in 1 sitting). Participants must complete their Sexual Health Essentials Core (enhanced or standard) before any remaining Cores in their randomized condition are made available. These remaining Cores may be completed in any order. Completed Cores may be revisited and reviewed as often as desired. While technical support is available from the UVA research coordinator, no clinical support will be provided. All Cores were written to an eighth-grade reading level (Flesch-Kincaid Grade Level) [47] or lower.

Participants will access their SHINE program via a password-protected website by computer, tablet, or smartphone. Participants will be monitored by the UVA research coordinator to ensure that they set up a password and can successfully log into the program. The goal of this monitoring is to ensure that the participants can access the intervention. Participants who do not complete their first log-in within a day of receiving the welcome email will receive daily automated reminder emails from the program. If the participant still has not logged in after 3 automated reminder emails, contacts will escalate to reminders by phone call and personal email by the UVA research coordinator and then by the local NCORP site research staff.

There are also automated email prompts to support participant engagement with the SHINE program after participants have successfully logged into the program. Participants who do not initiate Cores within 3 days of initial access will receive a reminder prompt. All participants will receive weekly progress emails regarding their progress through each of their assigned Cores. After participants complete their assigned SHINE program, email prompts will remind them that they may continue to access their SHINE program and review their Cores as desired.

### Measures

#### Overview

Study measures are summarized in Table 2. REDCap surveys require all items to be answered to limit missing data; however, participants may select a “refuse to answer” response for any item. Medical record data on enrolled survivors are collected by participating NCORP site research staff using a standardized medical record data extraction form in REDCap.

**Table 2:** Study measures.

Variable or construct			Measurement		Time point (weeks)				
					0 (baseline)		12		24
**Screening**									
	Cancer diagnosis and treatment; age	Medical record extraction							
	Sex and gender; relationship status; cancer-related sexual concerns; access to internet and email; serious mental illness; current therapy; and current pregnancy	Self-reported web-based screening questionnaire							
**Sample characteristics**									
	Demographics (eg, race, ethnicity, education, household income, sexual orientation, and partner gender)	Self-reported		✓					
	Medical history (eg, diagnosis, treatment type and dates, and other cancer history)	Medical record extraction		✓				✓	
	Recent sexual experiences, changes in partner status	Patient-Reported Outcomes Measurement Information System sexual functioning screener		✓		✓		✓	
**Sexual morbidity outcomes**									
	Sexual distress (primary)	Female Sexual Distress Scale-Desire, Arousal, Orgasm		✓		✓		✓	
	Sexual functioning (secondary)	Female Sexual Function Index		✓		✓		✓	
**Mediators**									
	Sexual functioning knowledge	4-item knowledge perception; 8-item knowledge quiz		✓		✓		✓	
	Sexual communication self-efficacy with clinicians	2-item confidence to raise and discuss sexual concerns		✓		✓		✓	
	Sexual communication self-efficacy with partner	Self-Efficacy to Communicate about Sex and Intimacy scale		✓		✓		✓	
	Emotional intimacy with their partner	Personal Assessment of Intimacy in Relationships—emotional intimacy subscale		✓		✓		✓	
**Moderators**									
	Menopause status	Self-reported		✓					
	Adjuvant endocrine therapy use	Medical record extraction		✓					
	Time since primary breast cancer treatment	Medical record extraction		✓					
	Psychological distress	Patient Health Questionnaire-4		✓		✓		✓	
	Relationship duration	Self-reported		✓					
	Relationship satisfaction	Relationship Assessment Scale		✓		✓		✓	
**Engagement and acceptability**									
	Engagement	Log-ins, Core completion (recorded by the intervention platform throughout the 24-week study period)							
	Satisfaction	Client Satisfaction Questionnaire-8				✓			
	Usability	Internet Intervention Utility Questionnaire				✓			

#### Sexual Morbidity Outcomes

Two outcomes will be evaluated to determine the optimal combination of intervention components: the Female Sexual Distress Scale–Desire, Arousal, Orgasm (FSDS-DAO [48,49]; primary outcome) and the Female Sexual Function Index (FSFI [50-52]; secondary outcome). These measures will be assessed at all 3 time points (ie, baseline, 12-week, and 24-week assessments); the primary evaluation is change between baseline and 24-week assessments.

The FSDS-DAO measures sexual distress among women and will be the primary outcome of the intervention optimization decision-making process. It has established reliability, construct and discriminant validity, and sensitivity to treatment-related changes [48,49]. Participants will rate 15 items on a 5-point Likert scale from 0 (never) to 4 (always). Total sum scores range from 0 to 60, with higher scores reflecting greater sexual distress.

The FSFI measures sexual functioning among women. It also has established reliability, construct and discriminant validity, and sensitivity to treatment-related changes [50-52]. Scores will only be evaluated from women who report having sexual activity within the past 4 weeks, as recommended [53]. Participants rate 19 items on a 5- or 6-point Likert scale from 0 (eg, no sexual activity) or 1 (eg, almost never or never) to 5 (eg, almost always or always). To compute a total score, summed scores of each of the 6 domains (desire, arousal, lubrication, orgasm, satisfaction, and pain) are scaled to a maximum score of 6, which are in turn summed for the total score. Total scores range from 2 to 30, with higher scores representing better sexual functioning and a total score of ≤26.0 suggestive of clinically relevant sexual dysfunction.

#### Mediators

The following a priori mediators will be tested: sexual functioning knowledge (knowledge perception and quiz questions, which were reviewed and revised by survivors of breast cancer); sexual communication self-efficacy with a health care provider (items on confidence to raise and discuss sexual concerns) [33]; sexual communication self-efficacy with partner (Self-efficacy to Communicate about Sex and Intimacy scale [54]); and emotional intimacy with partner (emotional intimacy subscale of the Personal Assessment of Intimacy in Relationships scale [55]). Mediator variables will be assessed at all 3 time points; the primary evaluation is change between baseline and 12-week assessments.

#### Moderators

The following a priori moderators will be collected or assessed at baseline: menopausal status (self-reported), use of AET (medical record extraction), time since primary breast cancer treatment completion (medical record extraction), psychological distress (Patient Health Questionnaire-4) [56], relationship duration (self-reported), and relationship satisfaction (Relationship Assessment Scale) [57]. Psychological distress and relationship satisfaction will also be measured at 12- and 24-week assessments as exploratory outcomes.

#### Engagement and Acceptability

Participants’ engagement with the SHINE intervention will be characterized by metrics including the number of log-ins and Core completion rates (collected automatically by the SHINE intervention platform). At the 12-week assessment, participants will rate their satisfaction with the SHINE intervention Cores (Client Satisfaction Questionnaire-8) [58] and perceived usability of the Cores (Internet Intervention Utility Questionnaire) [59].

#### Sample Size and Power Analysis

This factorial trial has been powered to detect a small-to-medium effect size (ie, Cohen *d*=0.30) of intervention components on sexual morbidity outcomes. This represents the smallest effect size that the study team determines would offset the required resources of an intervention component to be delivered to and completed by participants in an optimized intervention. Determined using the MOST R package [60] *FactorialPowerPlan* function (R version 3.6.2; R Foundation for Statistical Computing), 247 survivors of breast cancer would be needed to detect Cohen *d*=0.30 of change in sexual distress from baseline to 24-week assessment (measured by FSDS-DAO) at 80% power with α=.05 for all main effects and interaction effects of the intervention components. In a factorial experimental design with only 2 levels per factor, the power to detect main effects and interaction effects is equal [39,61]. This power analysis assumes a pretest-posttest correlation in sexual distress of *r*=0.65, which is supported by data from the investigators’ prior research (Female Sexual Distress Scale pretest-posttest *r*=0.73; N=28) [32] and by Female Sexual Distress Scale validation data (pretest-posttest *r*=0.62; N=318) [49]. The effect size of Cohen *d*=0.30 is reasonable to expect from intervention components, given that prior randomized controlled trials of interventions containing the components demonstrated large effects on sexual distress relative to control (Cohen *d*=0.87 and Cohen *d*=0.59) [28,32]. Conservatively accounting for an estimated 20% attrition (based on the investigators’ past trials) [32,42,62] and rounding up to achieve a balanced number of participants per condition, 320 survivors will be randomized in the trial (247/0.80=309 rounded to a total N=320, for n=20 per each of the 16 factorial conditions).

### Data Analysis Plan

#### Overview

In accordance with the NIH requirement, a Data Safety and Monitoring Plan has been established to guide study oversight and ensure the safety of participants and data validity and integrity. Summaries of primary and secondary measures will be reviewed for completeness every 6 months by the WF NCORP RB Data Safety Monitoring Board, along with other key study metrics such as recruitment, retention, patient characteristics, and adverse events. More details about trial oversight and monitoring are available in Multimedia Appendices 3 and 4.

Study methods have been designed to avoid missing data, including efforts to retain participants with incentives and assessment reminder protocols, as well as requiring responses to all items on web-based assessment questionnaires. As it is not possible to entirely avoid missing data, restricted maximum likelihood estimation will be used to address missing data in analyses under a missing at random (MAR) assumption [63-65]. Proposed analyses are robust against MAR data [63,64]. The National Academy of Sciences guidelines for missing data will be followed [64], and the impact of missing data on study conclusions will be explored with sensitivity analyses. Specifically, attrition rates across study conditions will be compared. If a particular condition has a markedly higher dropout rate, baseline covariates that predict attrition will be identified in secondary analyses. Variables determined to predict loss to follow-up will be included in sensitivity analysis models to satisfy the conditions described by Little and Rubin [65] for data to be considered MAR.

Primary analyses will be intention to treat, meaning that the randomized condition assignment will be included in models regardless of whether the participant completes their assigned Cores or not. Sensitivity analyses will examine the effects by whether assigned Cores are completed or not. All participants with at least 1 follow-up assessment will be included in analyses. All tests of hypotheses and reported P values will be 2 sided.

#### Primary Objective Analyses

The primary objective of this study is to identify the most efficient intervention package that is expected to produce the greatest benefit to survivors’ sexual health with the fewest (and least-intensive) intervention components. This objective will be achieved using an “all-active components” optimization criterion to guide a decision-making process with systematic data analysis [39]. The main and interaction effects of components will be computed using a generalized linear model (GLM) approach to ANOVA with effect coding to compare the average change in sexual morbidity aggregated across experimental conditions [66].

A model will be conducted for effects on sexual distress, measured by the FSDS-DAO; this model will be prioritized for the intervention optimization decision-making process. First, only components (and component levels) that have a beneficial independent effect (ie, a small-to-moderate positive main effect [Cohen *d≥*0.3]) on sexual distress will be provisionally included. Next, these provisional decisions will be reviewed by examining component interaction effects. Components will be considered for addition to the intervention package if they have important (ie, Cohen *d≥*0.3) synergistic effects, meaning they amplify the benefit of another component in the package. Components will be considered for removal from the package if they have important (ie, Cohen *d≥*0.3) antagonistic effects, meaning they reduce the benefit of another component in the package. Decisions will be informed by computing parsimonious prediction models for outcomes from both the main and interaction effects of the included components [39].

A subsequent model will be conducted for sexual functioning among women reporting sexual activity within the past 4 weeks, measured by the FSFI. Model results will be compared between sexual distress (full population) and sexual functioning (sexually active population only). Components (and component levels) provisionally included based on the sexual distress model findings may be removed if there is a substantial negative effect on sexual function. While discrepant or antagonistic results between the sexual distress and sexual functioning models are possible, they are unlikely, given that FSDS-DAO and FSFI total scores have been found to be highly correlated (*r=*–0.65 [49]).

#### Secondary Objective Analyses

Mediation and moderation will be examined separately and will follow the recommendations of prior work [66].

#### Mediation

An established strategy using structural equation modeling for testing mediation in a factorial experiment will be followed [67]. The mediation effects of components on both sexual distress and sexual functioning will be evaluated within a single model. Mediation hypotheses will be supported if (1) changes in the mediators influence the change in sexual morbidity, (2) intervention components influence the change in the mediators, and (3) changes in the mediators account for the relation between intervention components and changes in sexual morbidity.

In an optimization trial design, mediation analyses are critical both when components produce a positive effect and when there are null findings [68]*.* If there are null findings of components on primary outcomes, mediation analyses support the next research steps by determining where a breakdown may have occurred. For instance, a hypothesized mediator may influence the outcome, but the component did not adequately influence the mediator. This result would suggest that intervention revisions would be needed to better target the mediator. Mediator analyses are also critical to refining the study’s conceptual model.

#### Moderation

The effect of hypothesized moderators on the effects of intervention components on sexual morbidity will be tested by extending the GLM models from the primary objective analyses to include the interaction effects between intervention components and moderators [66]. Recommendations on centering continuous moderators (ie, time since treatment, psychological distress, and relationship duration and satisfaction) and effect coding categorical moderators (ie, menopausal status and AET use) will be followed [69]. Moderation hypotheses will be supported if the interaction effects are significant (P<.05). In addition to the limited set of a priori moderators, other potential moderators (eg, race, ethnicity) may be evaluated for hypothesis generation purposes, adjusting for multiple analyses using false discovery rates. As with mediation analyses, moderation analyses are important regardless of the component effects to understand whether particular groups of survivors differentially benefit from particular components (and combinations of components).

### Exploratory Analyses

We will examine survivors’ intervention engagement and acceptability. Means and SDs or frequencies, as appropriate, of user engagement metrics will be reported. Whether log-ins differ by components will be tested using the GLM approach as described for the primary objective analyses. The influence of engagement on the change in sexual morbidity outcomes will also be tested by extending GLM models from the primary objective analyses to include the interaction effects between intervention components and engagement. Users’ ratings of Core usability and satisfaction will also be reported.

## Results

This study is funded by the NCI (UG1CA189824 and R37CA269776; project duration: from January 2023 to December 2027). This trial is registered with ClinicalTrials.gov (NCT06216574). Data collection is planned to begin in March 2024 and conclude in 2027.

## Discussion

### Overview

Findings will be used to identify a breast cancer–related sexual health intervention package optimized for maximum impact by providing the greatest expected improvement with the fewest (and least-intensive) intervention components. This will be achieved by evaluating which sexual health intervention components meaningfully improve survivors’ sexual health either on their own or in combination with other components. Ensuring that only beneficial components are included in a resulting intervention eliminates unnecessary user effort. Minimizing the time burden to complete the resulting intervention is expected to limit a critical engagement barrier [28,30], permitting more survivors of breast cancer to benefit from this care.

More broadly, the factorial trial design will permit a rigorous evaluation of mechanisms of action for sexual health intervention components and suggest whether certain survivors may need distinct sexual health intervention approaches. These findings from our mediation and moderation analyses will fill a key gap in the literature regarding what specific intervention techniques work and for whom those techniques work best [29]. Furthermore, the results from this optimization trial could accelerate cancer-related sexual health intervention research by demonstrating which therapeutic components may best meet the specific needs, constraints, and characteristics of other target populations [70]. Indeed, the NIH recognizes rigorous testing of the mechanisms of behavioral intervention components as necessary to develop the next generation of more effective and efficient interventions [71].

### Comparison With Prior Work

Delivering the optimized and fully automated intervention via the internet promotes its potential impact through greater accessibility, as internet interventions are scalable and overcome many barriers to sexual health care in standard oncology practice. Although prior internet interventions for cancer-related sexual morbidity tested to date have included care delivered by a clinician [28,72-76], delivering such care without the need for clinician involvement would dramatically increase scalability and reduce long-term costs [77]. The fully automated delivery of care also has the advantage of being private, thereby removing the barrier of stigma related to discussing sex and giving women more control over their access to sexual health care after cancer [26,27,30]. Finally, fully automated interventions can be delivered with complete treatment integrity [78], ensuring each survivor using the program has a consistent experience.

### Limitations and Future Research

Future research steps are planned to address the limitations of this trial. In this trial, SHINE intervention components are only available in English. To increase the reach of this intervention, future transcreation work with Latina survivors of breast cancer is planned to both translate and culturally adapt the intervention to be appropriate for Spanish-speaking survivors [79-81]. In addition, this intervention only targets survivors of breast cancer and does not have content directed specifically for their partners. Future studies should evaluate whether providing companion educational materials to survivors’ partners may enhance intervention outcomes for couples. Finally, as our factorial trial is part of the *optimization phase* of the MOST research framework, a follow-on randomized controlled trial of the optimized intervention package will be needed to test the efficacy of this program.

### Conclusions

In summary, this novel optimization trial holds significant potential to address the critically undertreated and distressing breast cancer survivorship concern of sexual morbidity. The factorial trial design will begin to identify which sexual health intervention components work, for whom, and how. These data are directly responsive to calls from leaders in cancer control to advance intervention science through systematically testing intervention components and their mechanisms. By identifying the combination of the fewest and least-intensive intervention components that are likely to provide survivors the greatest benefit, this study will result in the first breast cancer–related sexual health internet intervention optimized for maximum population health impact.

## Appendix

Multimedia Appendix 1 National Institutes of Health grant reviews.

Multimedia Appendix 2 Sexual Health and Intimacy Enhancement Intervention details.

Multimedia Appendix 3 Sexual Health and Intimacy Enhancement oversight and monitoring details.

Multimedia Appendix 4 Data and safety monitoring plan.

## Abbreviations

AET: adjuvant endocrine therapy
CIRB: Central Institutional Review Board
FSDS-DAO: Female Sexual Distress Scale–Desire, Arousal, Orgasm
FSFI: Female Sexual Function Index
GLM: generalized linear model
HIPAA: Health Insurance Portability and Accountability
MAR: missing at random
MOST: multiphase optimization strategy
NCI: National Cancer Institute
NCORP: National Cancer Institute Community Oncology Research Program
NIH: National Institutes of Health
RB: Research Base
REDCap: Research Electronic Data Capture
SHINE: Sexual Health and Intimacy Enhancement
SPIRIT: Standard Protocol Items: Recommendations for Interventional Trials
UVA: University of Virginia
WF: Wake Forest

## Data Availability

Deidentified data sets used in primary publications will be transferred to the National Clinical Trials Network and National Cancer Institute Community Oncology Research Program Data Archive in accordance with the agreement between Wake Forest National Cancer Institute Community Oncology Research Program research base and the National Cancer Institute.
