# Supplementary material for: Factorial Trial to Optimize an Internet-Delivered Intervention for Sexual Health After Breast Cancer: Protocol for the WF-2202 Sexual Health and Intimacy Enhancement (SHINE) Trial
Source: JMIR Res Protoc. 2024 Aug 19;13:e57781. doi: 10.2196/57781 (PMC11369542; doi:10.2196/57781)
Supplement: Multimedia Appendix 1 [file resprot_v13i1e57781_app1.pdf]

Note: Content outside of reviews has been redacted throughout, but no modifications otherwise made

SUMMARY STATEMENT
(Privileged Communication) ***Release Date:*** **06/24/2022**
Revised Date:

Application Number: **1 R01 CA269776-01A1**
Principal Investigator ***Formerly:*** **1R01CA269776-01**

SHAFFER, KELLY MCLEAN

Applicant Organization: UNIVERSITY OF VIRGINIA

Review Group: **BMHO**
Biobehavioral Medicine and Health Outcomes Study Section

Meeting Date: **06/06/2022** ***RFA/PA:*** **PAR21-035**
Council: **OCT 2022** ***PCC:*** **MMOR**
Requested Start: **09/01/2022**

Project Title: **Optimizing psychosocial intervention for breast cancer-related sexual morbidity: A factorial trial using the National Cancer Institute Community Oncology Research Program (NCORP) network**

ADMINISTRATIVE BUDGET NOTE: The budget shown is the requested budget and has not been adjusted to reflect any recommendations made by reviewers. If an award is planned, the costs will be calculated by Institute grants management staff based on the recommendations outlined below in the COMMITTEE BUDGET RECOMMENDATIONS section.
EARLY STAGE INVESTIGATOR
NEW INVESTIGATOR

1R01CA269776-01A1 Shaffer, Kelly

RESUME AND SUMMARY OF DISCUSSION: This application proposes a mechanistic RCT of a multicomponent internet-based behavioral intervention to reduce women's sexual distress and dysfunction in breast cancer survivors. During discussion the panel agreed the significance of addressing sexual morbidity in this population is high, with potential to advance the field with an optimized scalable intervention. This productive and highly accomplished new investigator with her experienced investigative team were responsive to prior reviews, notably by providing additional support for their preliminary data, including moderators, adding a third Aim to evaluate engagement and acceptability of the intervention, and clarifying the feasibility of recruiting breast cancer survivors. Reviewers noted the use of the MOST design to optimize a full internet-based intervention without clinician involvement, the use of NCORP sites, and the use of mediator/moderator analyses of treatment effects to maximize care are innovative. Additional strengths include strong preliminary data supporting feasibility, a well-articulated conceptual model and fidelity monitoring protocol, detailed recruitment/retention plans and a rigorous study design. Reviewers also noted the adaption of the phone intervention to the internet intervention was not fully described and only 10 months were budgeted for its development and testing. Overall, the panel agreed the application will have a high impact on the field of breast cancer survivors with a scalable, low-touch intervention targeting sexual morbidity.

DESCRIPTION (provided by applicant): Approximately three-quarters of the 3.8 million breast cancer survivors in the U.S. experience clinically significant sexual distress and dysfunction. This cancer-related sexual morbidity can be addressed by Internet interventions that include multiple therapeutic techniques to target the overlapping biopsychosocial aspects of the problem; however, the full potential impact of these interventions has yet to be realized due to high refusal rates from time burden and high attrition rates. A more efficient sexual morbidity intervention that retains efficacy while lowering participant burden would have greater public health impact. Therefore, the goal of this proposal is to determine an Internet intervention that is optimized for greatest impact on breast cancer-related sexual morbidity, to evaluate mediators and moderators of intervention component efficacy, and examine survivors' engagement with the intervention components. Specifically, we will test four research-tested and fully-automated Internet intervention components for breast cancer survivors – psychoeducation about cancer-related sexual morbidity, training for communication with their clinicians, training for communication with their partners, and physical intimacy enhancement – using the Multiphase Optimization Strategy (MOST) research framework. We will recruit 320 intimately partnered women who have completed primary breast cancer treatment and report sexual morbidity through the NCI Community Oncology Research Program (NCORP). In the highly-efficient optimization trial, participants will be randomized to receive a combination of the four intervention components and will complete online assessments at baseline (pre-randomization), 3-month post-assessment, and 6-month post-assessment. For Aim 1, we determine the optimal breast cancer-related sexual morbidity intervention package by following a systematic data-driven approach that tests intervention component effects both independently and synergistically on sexual distress (primary outcome) and sexual functioning (secondary outcome). For Aim 2, we will evaluate mediators (sexual functioning knowledge; sexual communication self-efficacy with providers and with partners; relationship intimacy) and moderators (menopausal status at diagnosis; adjuvant endocrine therapy use; metastatic disease; psychological distress; relationship duration and dissatisfaction) of intervention component efficacy on sexual

morbidity. Last, for Aim 3, we will examine survivors' engagement and satisfaction with the intervention components. As the first MOST study of cancer-related sexual morbidity, this study is directly responsive to NOSI NOT-OD-20-106 and to calls from leaders in cancer control to advance intervention science through systematically testing intervention components and their mechanisms. By identifying the combination of sexual morbidity intervention components likely to provide breast cancer survivors the greatest benefit for the least burden, this study will result in the first Internet intervention optimized for maximum impact for the undertreated, prevalent, and devastating problem of breast cancer-related sexual morbidity.

PUBLIC HEALTH RELEVANCE: The proposed research is relevant to public health as about 1 in 8 women in the U.S. will be diagnosed with breast cancer in her lifetime, and about 3 in 4 of those women will experience clinically significant sexual morbidity. Relevant to the mission of the NCI, this study aims to improve cancer care by establishing an Internet intervention that provides breast cancer survivors the greatest sexual health benefit for the least burden. Findings will also improve cancer-related sexual morbidity intervention science by evaluating how intervention components work, for whom they work best, and how they can be made more engaging.

CRITIQUE 1

Overall Impact: This resubmission application proposes an MOST trial to test the main and interaction effects of fully automated internet intervention components in reducing sexual morbidity. Strengths include an early stage investigator with strong experience and a senior research team, addressing a significant but understudied problem, using the MOST design for optimization, accrual through NCORP, and intervention content were research tested. The resubmission mostly addressed previous reviewers' comments by including moderators in a comprehensive theoretical framework, adding a Aim 3 to evaluate the participant's engagement/usage of the intervention in relation to efficacy, clarifying the cognitive and behavioral skills in intervention component foundation, clarifying the capabilities of NCORP to recruit breast cancer survivors, and enhancing the rationale for optimization trial. However, the main concern is the Preparation phase of the MOST, that was not explicitly described and the feasibility of developing and adapting the intervention components in 10 months with pilot testing to support the Optimization phase. How to ensure intervention's fidelity when a phone-based intervention is adapted to an internet version is not clear. The very senior Co-I team with extensive Internet research lessen this concern. Overall, this is a strong application.

1. Significance:

Strengths

- Sexual health and function among cancer survivors is a significant problem but inadequately addressed.
- An optimized scalable intervention aimed to improve sexual dysfunction has the great potential to address this understudied problem.

- Preliminary studies of co-I's demonstrate the need to reduce participant's time burden, justifying the MOST approach.

Weaknesses

- None noted by reviewer.

2. Investigator(s):

Strengths

- PI is a highly accomplished early stage investigator.
- The research team has complementary expertise including clinical trials experience, MOST design, eHealth, outcomes research and sexual morbidity and interventions in breast cancer patients.

Weaknesses

- None noted by reviewer.

3. Innovation:

Strengths

- The application of MOST in the context of sexual morbidity among cancer patients is new.

Weaknesses

- Breast cancer population is the most studied cancer patient group, including for the research area of sexual morbidity.
- The delivery modality and format of the intervention component are not innovative, especially no engagement strategies (e.g. how to prompt participants to use the intervention) are elaborated.

4. Approach:

Strengths

- Recruitment via NCORP sites is a strength, potentially generate a diverse group of patients and improve generalizability of the study findings.
- Conceptual model clearly defines the proposed mediators, moderators, and outcomes.
- Intervention fidelity monitoring protocol was well articulated.
- Comprehensively evaluate engagement and usage in relation to intervention component efficacy is a strength.

Weaknesses

- The "Preparation" phase of the MOST, not explicitly described, is supposed to pilot test individual components. Although the intervention components will be derived from co-I's STC and IE interventions which are script- and phone-based, adapting two interventions into an internet delivery format including animation, video testimonials within 10- months during phase I seems very ambitious. And no pilot testing of the newly developed intervention components was described.

5. Environment:

Strengths

- UVA with very strong Internet research infrastructure, Fox Chase, and Wake Forest all have strong resources to support.

Weaknesses

- None noted by reviewer.

Study Timeline:

Strengths

- Very detailed.

Weaknesses

- The first 10 months of phase I includes intervention development, relatively ambitious if pilot testing is included.

CRITIQUE 2

Overall Impact: This study, led by an early-stage, new investigator, proposes to address the gap in clinical care of women's sexual distress and dysfunction after a diagnosis of breast cancer. The study proposes to use a MOST design to determine which components of a web-based, scalable intervention are most effective and for which women. Strengths of the study, beyond the MOST design and clinical significance, include evidence-based intervention components, a highly accomplished PI with the support of a strong investigative team, robust preliminary data, assessment of key mediators and moderators of treatment effects, and capacity to be highly scalable across treatment settings, especially those that are under-resourced. The notable weaknesses are (1) insufficient rationale for the selection of mediators and moderators in Aim 2, and (2) insufficient representation of racial/ethnic minority women in the sample to explore racial disparities in intervention effects. Although these are moderate weaknesses, the study is also very strong and has the potential for high impact on remediating a critical gap in current care of women with breast cancer.

1. Significance:

Strengths

- The significance of this study lies in addressing sexual distress and dysfunction among breast cancer survivors, which is a significant public health problem and not adequately addressed by current systems of cancer care.
- The premise of the study is that a scalable web-based intervention has the potential to mitigate the impact of sexual morbidity among women with breast cancer.

- While the effects of breast cancer diagnosis and treatment have been well-studied, suggesting that focus on another cancer might be preferable, the investigators support the need for intervention among this population, which has one of the highest rates of sexual morbidity.
- The web-based low-touch intervention has the potential for widespread impact across even under-resourced treatment settings.

Weaknesses

- The study is reportedly rooted in Social Cognitive Theory. However, the selection of particular mediators and moderators – a large piece in determining how and for whom interventions works – is not addressed. It is not clear if they are tied to the Social Cognitive Model or are supported by some other theoretical or empirical rationale.

2. Investigator(s):

Strengths

- The study PI (Shaffer), a highly experienced early-stage investigator, will have primary responsibility for all aspects of study implementation, quality assurance, staff oversight, data management and analysis oversight, and dissemination of results.
- Dr Shaffer is joined by experts at University of Virginia in female sexual dysfunction; program evaluation; internet interventions; breast oncology; study design including MOST trials; and instructional design and UI/UX.
- Study implementation is supported at additional sites (Wake Forest Health Sciences and Fox Chase Cancer Center) by site PIs (Drs. Danhauer and Reese).

Weaknesses

- None noted by reviewer.

3. Innovation:

Strengths

- First study to use MOST design to optimize a web-based intervention for maximum impact on the often overlooked and psychologically devastating problem of breast cancer-related sexual distress and dysfunction.
- The study will include mediator and moderator analyses to help maximize the individualized care available through the use of automated web-based intervention.
- The intervention is designed to address key barriers (time commitment and engagement).
- The study will yield isolated understanding of treatment receipt and treatment engagement.
- First study to use NCORP sites for an optimization trial.

Weaknesses

- None noted by reviewer.

4. Approach:

Strengths

- Use of a multiphase optimization trial (MOST) design provides methodological rigor to determine optimal components of the internet-based intervention.

- Detailed and robust recruitment and retention plan.
- Women will be randomized to combination of four intervention components that have been selected based on prior research (psychoeducation, communication training with clinicians, communication training with partners, and physical intimacy promotion).
- A web-based intervention allows for added features, such as animation, video, and automated email prompts, as well as ensures consistency in the delivery of content.
- Analysis of mediators and moderators will allow exploration of factors that may make the intervention more effective in some women relative to others.
- The study will incorporate an aim to determine survivor engagement and acceptability.

Weaknesses

- The representation of women of diverse racial/ethnic minorities is proposed to be 34% (66% Non-Hispanic white) in this sample to be in line with previous studies conducted through the Wake Forest NCORP Research Base. However, with over 21,000 women eligible, this is a missed opportunity to achieve racial/ethnic representation closer to (or exceeding) the national population. The ability to understand racial disparities in sexual morbidity – and post-intervention improvement – between white and non-white women with breast cancer seems a critical piece of intervention development that is missed in this study.

5. Environment:

Strengths

- The institutional environment and resources at the three study sites (University of Virginia, Wake Forest Health Sciences, and Fox Chase Cancer Center) will provide necessary support for the successful implementation of the study.

Weaknesses

- None noted by reviewer.

CRITIQUE 3

Overall Impact: This R01 resubmission from NI/ESI Shaffer uses a Multiphase Optimization Strategy trial design to determine the optimal intervention package for breast cancer-related sexual morbidity, as well as to describe *how* and for *whom* intervention components work best, and intervention engagement and acceptability. The application is noted to be *highly* responsive to prior critiques with several substantial modifications to the approach that address prior concerns about interdependence of aims and increase potential impact of study findings. The significance of the public health problem addressed and the potential for this application to contribute substantially to understanding in the field are high. The NI/ESI PI, Shaffer, has an outstanding track record of productivity, having secured a R21 award as PI since the last proposal, and support from the experienced and collaborative team is evident throughout the application. Several rigorous and innovative aspects to the approach are noted, and overall it was difficult to find weaknesses in this revision. The potential impact is deemed to be high.

1. Significance:

Strengths

- Sexual morbidity among breast cancer survivors is a highly significant public health problem and consistently rated as a distressing problem by patients.
- A sexual health intervention that could be successfully delivered via the internet and with high engagement would have a high impact.
- The application includes a review of the literature supporting this Multiphase Optimization Trial as the next step in this line of research likely to yield the most significant results.

Weaknesses

- None noted by reviewer.

2. Investigator(s):

Strengths

- Dr. Shaffer is a highly accomplished early-stage investigator with 45 pubs, recently received a R21 as PI.
- Investigative team has a track record of collaboration, extensive experience in clinical trials, behavioral interventions, and sexual health. They are collectively invested in the success of the project and in supporting the early-stage PI, Dr. Shaffer.
- Co-I Ritterbrand is Dr. Shaffer’s primary mentor.
- Team includes a breast surgeon and a biostatistician.

- Dr Collins as a consultant and MOST expert is a strength – letter of support evidences her investment in this revised application.

Weaknesses

- None noted by reviewer.

3. Innovation:

Strengths

- The optimization trial design is innovative in this context and allows for studying the receipt and impact of individual intervention components.
- A fully internet-based sexual health intervention that can be delivered without clinician involvement is innovative.
- Use of the NCI community oncology research program network is innovative and enhances external validity.
- Inclusion of women with metastatic breast cancer is innovative in a sexual morbidity study.

Weaknesses

- None noted by reviewer.

4. Approach:

Strengths

- The approach is based on rigorous research supporting the team's ability to successfully deliver and evaluate internet-based interventions in this population, as well as the basis for the 4 intervention components.
- There is a strong conceptual model included.
- NCORP sites will support robust recruitment, plans to achieve ≥ 33% from racial/ethnic minority backgrounds.
- Outcome measures, mediators and moderators are psychometrically sound and well-justified.
- Several rigorous aspects to the study design, including plans for building intervention components into an existing platform, plans for blinding participants and outcome assessments, appropriate power calculations accounting for attrition, and recruitment plans.
- Plans for assessing medicators, moderators and engagement will yield important insights regardless of trial outcomes.

Weaknesses

- Stage IV patients will not be ≥ 3 months from treatment with curative intent – this would seem to be a contradiction in inclusion criteria.

5. Environment:

Strengths

- Robust research environment and facilities at UVA to support internet based behavioral intervention trials.

- NCORP collaboration is a strength. Includes letters of support from participating NCORP sites to support recruitment goals.

Weaknesses

- None noted by reviewer.

Study Timeline:

Strengths

- Appropriately includes and justifies initial phase for NCORP set-up and study preparation.
- Includes review of recruitment process after 45 participants enrolled.
- Includes adequate time for analyses and follow-up R01 development.

Weaknesses

- None noted by reviewer.
