# Supplementary material for: Factorial Trial to Optimize an Internet-Delivered Intervention for Sexual Health After Breast Cancer: Protocol for the WF-2202 Sexual Health and Intimacy Enhancement (SHINE) Trial
Source: JMIR Res Protoc. 2024 Aug 19;13:e57781. doi: 10.2196/57781 (PMC11369542; doi:10.2196/57781)
Supplement: Multimedia Appendix 2 [file resprot_v13i1e57781_app2.pdf]

Appendix to: Shaffer, Reese, Dressler, et al. (2024) Factorial trial to optimize an internet-delivered intervention for sexual health after breast cancer: Protocol for the WF-2202 SHINE trial. JMIR Res Protoc. doi: 10.2196/57781

Multimedia Appendix 2. Sexual Health and Intimacy Enhancement (SHINE) Intervention Details

Component	Sexual Health Essentials*		Health Care Discussions	Partner Conversations	Intimacy Insights
Core	Standard	Enhanced			
Purpose	Increase survivors' understanding of women's sexual health and how cancer and cancer treatment can affect sexual health		Increase survivors' skills and confidence to raise and discuss cancer-related sexual concerns with their health care providers	Increase survivors' skills and confidence to raise and discuss cancer-related sexual concerns with an intimate partner	Increase survivors' physical and emotional closeness with their intimate partner
Learning objectives (as presented in the Core)	1. Discover how one's body, mind, and relationship are related to sexual health<br>2. Identify the different parts of the vulva<br>3. Recognize how the body and mind respond during sexual intimacy<br>4. See how cancer treatments can affect sexual health<br>5. Explore different strategies for managing sexual concerns<br>6. Pinpoint strategies for managing your sexual concerns		1. Recognize the importance and challenges of being engaged in your health care for sexual health<br>2. Plan ahead for effective conversations about sexual concerns with your health care team<br>3. Prepare effective ways of starting discussions about sexual concerns with your health care team using a structured communication model<br>4. Identify and prioritize your sexual concerns to discuss with your health care team<br>5. See what steps to take following a discussion with your health care team<br>6. Anticipate and plan for communication challenges with your health care team	1. Distinguish different types of communication (sharing, problem-solving, and non-verbal)<br>2. Recognize ineffective and effective communication practices<br>3. Explore how to open the door to more frequent and effective sexual communication in your relationship<br>4. Discover how to use effective communication tools to increase understanding between you and your partner<br>5. Prepare to follow a step-by-step problem-solving process together with your partner to address a sexual concern<br>6. Anticipate and plan for communication challenges with your partner	1. Specify your personal definition of intimacy in your relationship<br>2. Discover how to build intimacy with your partner through different kinds of activities<br>3. Define how your thoughts can influence intimacy in your relationship<br>4. Challenge unhelpful thoughts that get in the way of intimacy<br>5. Develop the skill of staying in-the-moment during physical intimacy by focusing on sensations<br>6. Anticipate and plan for intimacy challenges
Units and content summary	1. Introduction: Defining sexual health; biopsychosocial model of sexual health; normalizing experience of sexual concerns after cancer<br>2. Body, mind, and intimacy: Women's sexual anatomy review; sexual response cycle<br>3. Sexual health after breast cancer: Role of estrogen in women's sexual health; influence of breast cancer treatments on sexual health<br>4. Managing common concerns: Strategies for managing pain with sex, low sexual desire, low body image, and orgasm difficulty<br>5. Next steps: (Enhanced only: Coping plan activity); Core summary		1. Introduction: Defining healthcare engagement; normalizing challenges to raising sexual concerns with providers<br>2. Before the discussion: Common barriers preventing discussions; identifying which provider to approach<br>3. Having the discussion: SEA (be Specific, Explain why the issue is important, Ask a question) model of communication<br>4. After the discussion: Following up effectively after health care discussions<br>5. Planning for your discussion: Activity to prepare own SEA phrase and discussion<br>6. Next steps: Coping plan activity; Core summary	1. Introduction: Defining communication and non-verbal communication; normalizing couples' difficulties discussing sexual concerns after cancer<br>2. Tools for effective conversations: Behaviors for effective speaking and listening<br>3. Preparing for an effective conversation: Planning for conversations by clarifying goals and preparing an opening phrase<br>4. Sharing conversations: Defining and examples of sharing conversations<br>5. Problem-solving conversations: Defining problem-solving process and conversations<br>6. Next steps: Coping plan activity; Core summary	1. Introduction: Defining physical and emotional intimacy; normalizing intimacy challenges in relationships<br>2. Opening up to change: Link between thoughts, feelings, and intimacy behaviors<br>3. Thinking differently for intimacy: Defining maladaptive thinking and cognitive restructuring; cognitive restructuring activity<br>4. Focusing on intimacy: The rationale for and practice of sensate focus<br>5. Next steps: Coping plan activity; Core summary
Interactive features	None	3 Interactivities; 3 Videos; Content tailoring (Units 3 & 4)	2 Interactivities; 2 Videos	2 Interactivities; 3 Videos; 4 Audio files	3 Interactivities; 1 Video; 1 Audio file

*Participants are randomized to receive either the Standard or Enhanced Sexual Health Essentials Core; participants must complete their Sexual Health Essentials Core before any remaining assigned Cores are made available.
