# Supplementary material for: Factorial Trial to Optimize an Internet-Delivered Intervention for Sexual Health After Breast Cancer: Protocol for the WF-2202 Sexual Health and Intimacy Enhancement (SHINE) Trial
Source: JMIR Res Protoc. 2024 Aug 19;13:e57781. doi: 10.2196/57781 (PMC11369542; doi:10.2196/57781)
Supplement: Multimedia Appendix 3 [file resprot_v13i1e57781_app3.pdf]

Appendix to: Shaffer, Reese, Dressler, et al. (2024) Factorial trial to optimize an internet-delivered intervention for sexual health after breast cancer: Protocol for the WF-2202 SHINE trial. JMIR Res Protoc. doi: 10.2196/57781

Multimedia Appendix 3. SHINE Oversight and Monitoring Details

Data Category	Information
**Composition of the data monitoring committee, its role and reporting structure**	The Data and Safety Monitoring Board (DSMB) includes members demonstrating experience and expertise in oncology, biological sciences, biostatistics, and ethics and meets twice a year to assess all WF NCORP RB studies. The DSMB report is generated by the WF NCORP RB statistician. Reports of all DSMB meetings and recommendations will be provided to the NCI, CIRB, WF NCORP RB, and participating sites, as requested.
**Adverse event reporting and harms**	The descriptions and grading scales found in the revised NCI Common Terminology Criteria for Adverse Events (CTCAE) version 5.0 will be utilized for AE reporting. The possibility that the AE is related to study agent or intervention will be classified as one of the following attributions: Unrelated, Unlikely, Possible, Probable, Definite. Only unexpected grade 3, 4 or 5 AE that are deemed to be possibly, probably, or definitely related to the specific use of the SHINE intervention (from randomization through the 24-week time point) will be captured for this study. Reportable events as described above will be reported to the WF NCORP RB using the AE Reporting form in REDCap. Serious AE should be entered into REDCap within 10 calendar days of learning of the event. Site staff and/or Principal Investigators will also notify WF NCORP RB via REDCap reporting within 24 hours of discovering the details of any severe, life-threatening (grade 3 or 4) or fatal AE (grade 5) that are unexpected and deemed to be possibly, probably, or definitely related to specific use of the SHINE intervention. All reportable AE submitted to the WF NCORP RB will be reviewed by the WF NCORP RB Data and Safety Monitoring Committee (DSMC) and will be included in statistical reports to the WF NCORP RB DSMB.
**Frequency and plans for auditing trial conduct**	The WF NCORP RB DSMC meets monthly to review reportable AEs and Protocol Deviations to identify urgent safety and data concerns that may affect study safety and data quality. AE and Protocol Deviation reports are generated by the WF NCORP RB Data Management team. The DSMC consists of members of the WF NCORP RB team including one of the NCORP Research Base MPIs, the WF NCORP RB Administrator, regulatory, and data team members.

	The WF NCORP RB is also required to audit NCORP Community sites that have accrued any participants to WF NCORP trials at least once every 36 months. The audit assess local site regulatory compliance, confirms eligibility of enrolled participants, ensures all study activities are followed per protocol and reviews data accuracy and quality. A minimum of 10% of cases are audited per site.
