# Supplementary material for: Factorial Trial to Optimize an Internet-Delivered Intervention for Sexual Health After Breast Cancer: Protocol for the WF-2202 Sexual Health and Intimacy Enhancement (SHINE) Trial
Source: JMIR Res Protoc. 2024 Aug 19;13:e57781. doi: 10.2196/57781 (PMC11369542; doi:10.2196/57781)
Supplement: Multimedia Appendix 4 [file resprot_v13i1e57781_app4.pdf]

Appendix to: Shaffer, Reese, Dressler, et al. (2024) Factorial trial to optimize an internet-delivered intervention for sexual health after breast cancer: Protocol for the WF-2202 SHINE trial. JMIR Res Protoc. doi: 10.2196/57781

Multimedia Appendix 4. Data and Safety Monitoring Plan

In accordance with the NIH requirement, a Data Safety and Monitoring Plan (DSMP) has been established to guide the oversight of this study in order to ensure the safety of participants and the validity and integrity of the data. This monitoring will be commensurate with minimal risk present to participants.

The Wake Forest NCORP Research Base Data and Safety Monitoring Committee (DSMC) meets monthly to review reportable Adverse Events and Protocol Deviations to identify urgent safety and data concerns that may affect study safety and data quality. Adverse Event and Protocol Deviation reports are generated by the Wake Forest NCORP Research Base Data Management team. The DSMC consists of members of the Wake Forest NCORP Research Base team including one of the NCORP Research Base Multi-PIs, the Wake Forest NCORP Research Base Administrator, regulatory, and data team members.

The Wake Forest NCORP Research Base Data Safety Monitoring Board meets every six months to review Wake Forest NCORP Research Base protocols. The Board includes members demonstrating experience and expertise in oncology, biological sciences, biostatistics, and ethics. The DSMB report is generated by the Research Base statistician. Areas of review may include the following: Study Objectives; Patient Accrual; Patient Status and Retention; Study Status; Last Contact Status; Patient Compliance; Number of Biopsies/Labs as needed; Patient Characteristics; Summary of Observed Toxicities; Adverse Events; Date, Event briefly described Relationship to Drug, condition assigned; Summary of Primary and Secondary Measures. PRO-CTCAE data should not be used in adverse event stopping rules.

DSMB Responsibilities: The DSMB reviews accrual information and interim analyses of outcome data and cumulative toxicity data summaries to determine whether:

- the trial should continue as originally designed
- the trial should be changed
- the trial should be terminated
- outcome results should be released prior to the reporting of the study results

Members of this committee as well as the organization statistician will oversee the safety monitoring of the study to ensure that the privacy of all participants in the study is protected; ensure that participants' interests are primary, that is, above the interests of the scientific investigation; and to ensure that all data collection is scrutinized for accuracy, privacy and levels of protection. The committee will perform reviews of the data handling and confidentiality and comply with recommendations to resolve such problems and maintain written communication of the deliberations and recommendations that arise from their meetings. By examining this information, they will keep abreast of critical issues regarding recruitment and data integrity. Reports of all DSMB meetings and recommendations will be provided to the NCI, CIRB, WF NCORP RB, and participating sites, as requested.
